# Retraction Note: The distribution and socioeconomic burden of Hepatitis C virus in South Australia: a cross-sectional study 2010–2016

**DOI:** 10.1186/s12889-021-10978-4

**Published:** 2021-06-02

**Authors:** Bernard Luke Edmunds, Emma Ruth Miller, George Tsourtos

**Affiliations:** Flinders University, GPO Box 2100, Adelaide, 5001 South Australia

**Retraction to: BMC Public Health 19, 527 (2019)**

**https://doi.org/10.1186/s12889-019-6847-5**

**Retraction Note**

The Editor has retracted this Article at the request of the authors. After publication, it was realised that the sub-section of the secondary analysis that specifically focused on the de-identified data related to Indigenous notifications was not included in the original protocol approved by the SA Department for Health and Wellbeing Human Research Ethics Committee. While subsequent investigations by Flinders University found this to be due to an honest oversight, which was also concluded by the SA DHW Ethics Committee, it was requested that the article be retracted due to the unintended breach of the approved protocol. All authors agree to this retraction.

